# Screening for Dysglycemia: Connecting Supply and Demand to Slow Growth in Diabetes Incidence

**DOI:** 10.1371/journal.pmed.1002084

**Published:** 2016-07-19

**Authors:** Mohammed K. Ali, K. M. Venkat Narayan

**Affiliations:** Emory Global Diabetes Research Center, Hubert Department of Global Health, Rollins School of Public Health, Emory University, Atlanta, Georgia, United States of America

## Abstract

Mohammed Ali and Venkat Narayan describe the challenge of implementing evidence-based interventions for prevention for the large number of people at increased risk of type 2 diabetes.

Diabetes is one of the most devastating and costly conditions worldwide, leading to substantial burdens of macro- and microvascular diseases, as well as other disorders. Armed with evidence from randomized controlled trials [1–3] and other data showing that progression to type 2 diabetes can be effectively [4] and cost-effectively [5] delayed among people at high risk, several countries have embarked on rolling out prevention programs to slow the growing incidence of diabetes. Such programs center on evidence-based behavior change interventions aimed at promoting healthy diets, appropriate physical activity, and modest weight loss. The most recent initiatives include the United States government’s authorization for diabetes preventive services to be covered for Medicare beneficiaries [6] and the launch by the United Kingdom’s National Health Service of a nationwide diabetes prevention program [7]. However, for these endeavors to successfully mitigate growing diabetes burdens, several important barriers to implementation of prevention strategies need to be overcome (Fig 1), and screening for dysglycemia is a key part of this process to connect demand with a growing supply of preventive services.

**Fig 1:**
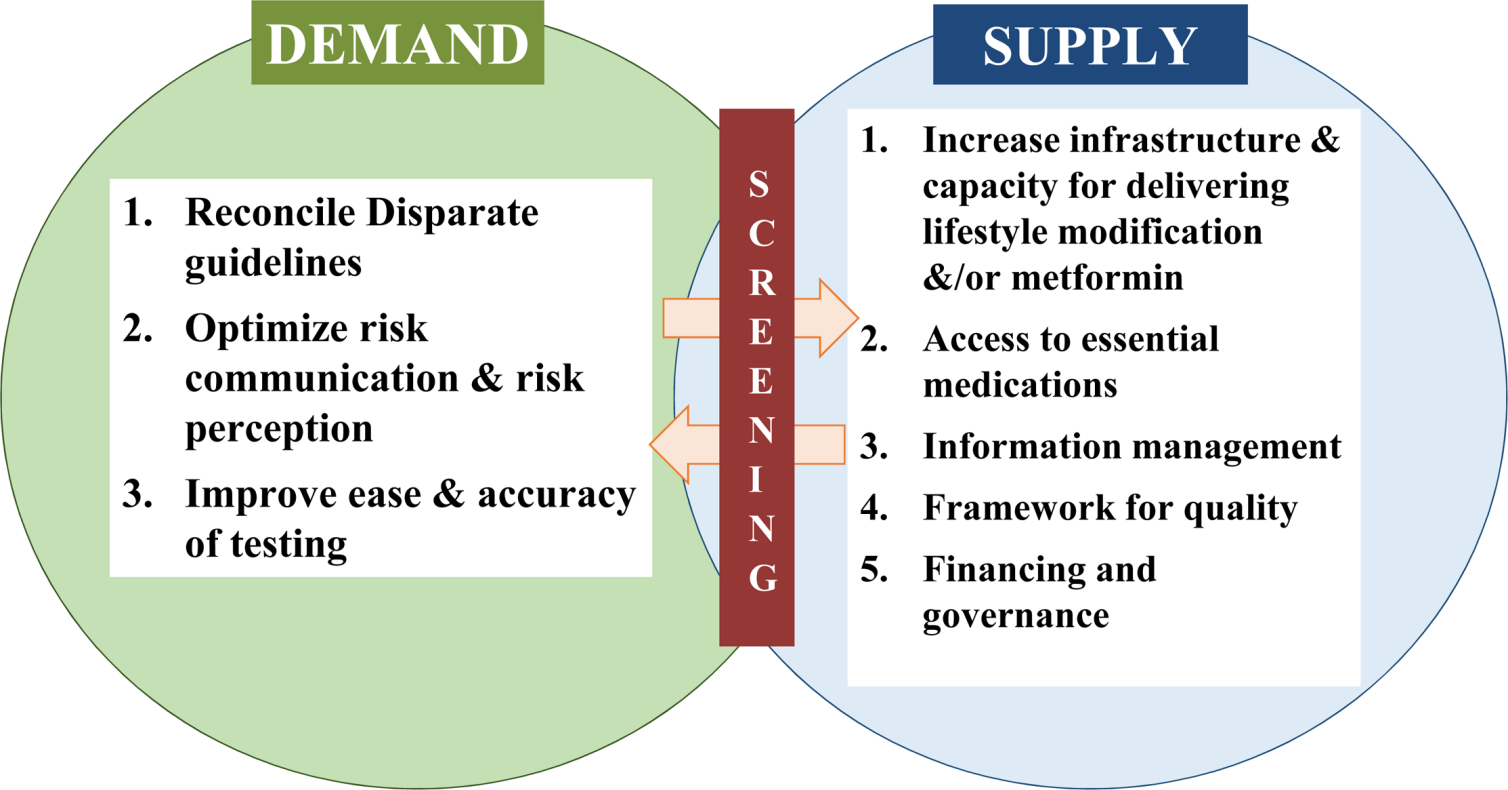
A framework for enhancing diabetes prevention and management supply and demand.

The principle that underlies screening for dysglycemia is to accurately identify risk of type 2 diabetes without causing physical or psychological harm and to motivate at-risk individuals to connect with appropriate health care and preventive services. While definitions of prediabetes vary, people with elevated fasting or 2-hour glucose, hemoglobin A1c, or any combination of these, albeit not in the diabetes range, have a 4–12 times higher annual likelihood of developing diabetes than the general, normoglycemic population [8]. Screening is therefore pertinent to identify these persons, especially since nearly 47% of the 415 million people with diabetes worldwide, and a substantial yet unquantified proportion of those with prediabetes, remain unaware of their condition [9]. Even in high-income countries like the US, over a quarter of the 29 million Americans with diabetes [10] and nearly 90% of the 86 million with prediabetes are not aware of their condition [11]. These awareness gaps are likely to impede success of evidence-based interventions to prevent diabetes and its complications among adults with both prediabetes and diabetes.

Although there are well-accepted, minimally-invasive glucose tests to accurately identify people with diabetes and prediabetes, and broad agreement that targeting persons at high risk for diabetes and offering glucose testing is more cost-effective and less harmful than universal testing [12], screening for dysglycemia has remained a contentious public health proposition for almost two decades. Debates about whether or not, and in whom, to encourage screening have led to disparate guidelines from influential expert committees [13,14], which may have contributed to large gaps in receipt of testing among those eligible. Indeed, recent national data from the US showed that only half of those eligible by the US Preventive Services Task Force (USPSTF) or the American Diabetes Association (ADA) guidelines reported having a glucose test in the past 3 years [15]. Harmonizing guidelines regarding glucose testing for high-risk individuals will likely be important in ensuring that people with dysglycemia are aware of their status and that appropriate services are available. The recent expansion of criteria for dysglycemia screening, to adults aged 40–70 years who are overweight or obese, by the USPSTF is a welcome step [13]. However, the criteria still exclude normal-weight people with dysglycemia, a group that may be especially prevalent in some populations, such as those of Asian or sub-Saharan African ancestry. Furthermore, since demand for and use of preventive services has been suboptimal to date, improving perception of and communication about risk in a way that motivates engagement will help to ensure that there is appropriate demand for the supply of preventive and health care services being created.

However, addressing low demand and engagement alone are not sufficient to address the problem of type 2 diabetes. Several enhancements are needed to the supply of diabetes care and preventive services. Of note, screening leads to detection of both undetected prediabetes and diabetes, and considering this continuum of risk could motivate greater integration of prevention and care—avoiding silos within diabetes would be more efficient and may promote continuity of care. In terms of diabetes care, a large proportion of people diagnosed with diabetes do not meet their care goals [10]; the absolute numbers of patients affected by disabling complications has increased; costs of care double every decade; and, although excess mortality associated with diabetes has declined for individuals in high-income countries [16], there has been an increase in the number of years lived with disability among people with diabetes. Therefore, appropriate public health policy, optimizing care services through the use of quality improvement mechanisms, information management and accountability, and patient-centered care delivery are all needed to manage the growing number of people with and costs of diabetes [17].

Supply-side concerns also include gaps in access, suboptimal organization and delivery of health care and prevention services, and workforce shortages. For prevention, for example, current infrastructure and capacity (e.g., to effectively deliver proven lifestyle interventions) are in far shorter supply in the US than the need suggests [18]. In addition to this, though data suggest that maintaining a healthy weight after initial weight loss further reduces diabetes incidence [19], most delivery programs have short-term maintenance components, and no approach has been endorsed as a minimum standard. Also, although the announcement that Medicare will finance diabetes preventive services in the US is momentous [6], the incidence of diabetes among non-Medicare-eligible young and middle-aged adults is not insubstantial; in fact, diabetes incidence among 45–64 year olds is almost the same as in those aged 65 and older [20]. Consequently, a wider array of financing options (e.g., through employers or safety nets) are needed, especially for people of lower socioeconomic status and minority populations who tend to be at the highest risk of diabetes [21]. An important consideration for countries with market-based health care like the US is who should pay for preventive interventions if the individual is unable to do so, and indeed another concern is whether lack of coverage for preventive services leads to greater health disparities.

Other considerations in successfully rolling out diabetes preventive services include governance, a framework for quality measurement, and offering different options for prevention, including the use of metformin for people with prediabetes. The US Centers for Disease Control and Prevention have created a set of quality assurance criteria through the Diabetes Prevention Recognition Program [22], which certifies providers nationally. In addition to this, establishing a set of aspirational, yet pragmatic, quality indicators (e.g., proportion of screen-eligible people tested, proportion of people with prediabetes referred to certified lifestyle programs, and proportion of those enrolled in lifestyle programs attending a minimum number of sessions and/or achieving behavior changes), as was done successfully for diabetes care in the 1990s, may have important impacts on process and health outcomes. These measures would also help stimulate governance structures, real-time quality improvement opportunities, and systems to drive better prevention services. Indeed, with the momentum of greater political will and efforts to optimize the balance of supply and demand for lifestyle modification programs underway in the US, it is also time for the broader diabetes community to collectively support the use of metformin for prevention. There is sufficient evidence to support its use [1], with few concerns about harm that should prevent action.

In a complex, fast-changing world, diabetes prevention offers a strategic opportunity to achieve better health at lower cost for a large proportion of the population [17]. It is important to note that the goal of scaling-up supply and demand of preventive and care services for high-risk individuals should be considered complementary to—and not mutually exclusive with—society-level population-based policies that need to advance in terms of rigor of the evidence base. However, to successfully achieve the aspiration of ubiquitous diabetes prevention, we need to harness all of the evidence-based intervention options at our disposal, build the systems and governance needed to optimize delivery of care and prevention, and, most importantly, rally behind a single, aligned set of screening guidelines to identify and connect supply with demand.

## Abbreviations

ADA: American Diabetes Association
USPSTF: United States Preventive Services Task Force
